# Clinical and functional effectiveness of anti-TNF therapy in patients with rheumatoid arthritis

**DOI:** 10.1186/1479-5876-9-S2-P46

**Published:** 2011-11-23

**Authors:** Rosalia Martínez-Pérez, Julia Uceda, Mario León, Sergio Rodríguez, Alejandro Muñoz, Francisco Gallo, Maria Luisa Velloso, Jose Luis Marenco

**Affiliations:** 1 Rheumatology Unit, Valme University Hospital, Sevilla, Spain

## Objective

To evaluate the clinical and functional activity of patients with rheumatoid arthritis at one and two years after starting anti-TNF therapy.

## Patients and methods

This is a retrospective study of 50 RA patients, who started ANTI-TNF theraphy between June 2006 and july 2009. The main variables in the study were: DAS28, HAQ and pain VAS. We analyzed evolution after 1 year and 2 years from the beginning of the treatment. The comparison with the basal state was made with Student’s *t* test. The statistical study was analyzed by the SPSS 18,0.

## Results

The study includes 50 patients, 66% female, with RF in 76% and erosions in 64%, treated with etanercept (28), adalimumab (13) and infliximab (9). Most of them (70%) had recieved at least 2 FAMES before biological. In the below table we compare changes in clinical parameters for different biologics after one and two years.

**Table 1:** 

	BASAL	12 MONTHS (p basal-12M)	24 MONTHS (p 12M-24M)
EVA (mm)			
-Etanercept	72,21±16,78	33,20 ± 20,05 (p<0,005)	31,95 ± 27,12 (p=0,39)
-Adalimumab	69,83±20,54	45,00 ± 28,33 (p=0,21)	51,00 ± 28,44 (p=0,16)
-Infliximab	69,78±15,45	42,56 ± 23,41 (p=0,015)	37,71 ± 23,95 (p=0,86)

DAS28			
-Etanercept	5,88 ± 1,05	2,85 ± 1,16 (p<0,005)	3,09 ± 1,52 (p=0,98)
-Adalimumab	5,73 ± 1,51	4,05 ± 2,43 (p=0,011)	3,90 ± 1,88 (p=0,22)
-Infliximab	6,03 ± 1,87	4,08 ± 1,61 (p=0,021)	3,83 ± 1,66 (p=0,91)

HAQ			
-Etanercept	1,61 ± 0,61	0,77 ± 0,64 (p <0,005)	0,80 ± 0,73 (p = 0,87)
-Adalimumab	1,63 ± 0,58	1,35 ± 0,63 (p = 0,05)	1,38 ± 0,70 (p =0,58)
-Infliximab	1,45 ± 0,74	1,24 ± 0,72 (p = 0,20)	1,28 ± 0,69 (p = 0,34)

## Conclusions

All anti-TNF significantly improve disease activity, functional status and pain VAS after the first year. This improvement continues but not significantly increases the second year.

Etanercept is the only drug with a significant reduction for HAQ, DAS28 and pain VAS.

